# Microwave-assisted pyrolysis of pine sawdust: Process modelling, performance optimization and economic evaluation for bioenergy recovery

**DOI:** 10.1016/j.heliyon.2023.e14688

**Published:** 2023-03-20

**Authors:** Denzel Christopher Makepa, Chido Hermes Chihobo, Walter Rutendo Ruziwa, Downmore Musademba

**Affiliations:** Department of Fuels and Energy Engineering, School of Engineering Sciences and Technology, Chinhoyi University of Technology, Private Bag 7724, Chinhoyi, Zimbabwe

**Keywords:** Biomass pyrolysis, Aspen Plus®, Response surface methodology, Bio-oil production, Optimization

## Abstract

This study aims at optimizing the process conditions to extract maximum yields of bio-oil from pine sawdust using microwave-assisted pyrolysis (MAP). Aspen Plus® V11 was used to model the thermochemical conversion of pine sawdust to pyrolysis products, and response surface methodology (RSM) based on a central composite design (CCD) was employed in the optimization of the process parameters. The mutual effects of pyrolysis temperature and reactor pressure on the product distribution were investigated. The findings have shown that the optimal operating conditions for producing the highest amount of bio-oil (65.8 wt%) were achieved at 550 °C and 1 atm. The product distribution of the simulated model was more significantly influenced by linear and quadratic terms of the reaction temperature. In addition, a high determination coefficient (R^2^ = 0.9883) was obtained for the developed quadratic model. A set of three published experimental results acquired under circumstances comparable to the simulations' operating limitations were used to further validate the simulation results. The process’s economic viability was assessed in order to establish the bio-oil minimum selling price (MSP). A MSP of $1.14/L of liquid bio-oil was evaluated. An economic sensitivity analysis has shown that the annual fuel yield, required rate of return, annual income tax, annual operating costs and initial capital investment have a substantial impact on the MSP of bio-oil. It was inferred that using the optimized process parameters may improve the process' competitiveness on an industrial scale due to its better product yields and improved sustainability in biorefineries, as well as assure waste reduction.

## 1 Introduction

Due to the rapid increase of the world’s population and the emergency of new technologies, the energy demand is rising rapidly, while the reserves of the currently available energy supplies are depleting [1]. The relevance of renewable and sustainable energy sources is simultaneously raised by environmental concerns and a potential crisis in energy production and sustainability [2]. Biomass has received a lot of attention recently amongst other renewable energy resources since it is the only renewable source of fixed carbon [3].

Each year, a large amount of forestry and agro-industrial waste is dumped; nevertheless, it can be collected and transformed into energy using thermochemical or biological conversion techniques [4]. The Zimbabwe timber industry depends on timber plantations located in the Eastern Highlands, occupying about 0.025% of dry land in Zimbabwe, comprising 130, 000, 000 m^2^ of wattle, 240, 000, 000 m^2^ of eucalyptus, and 810, 000, 000 m^2^ of pine. The industry generates over 70,000 tons of pine sawdust every year with an energy potential estimated at 232 kt_Oe_ [5]. The quantity of pine sawdust produced is anticipated to double in the next five years due to rapid industrialization and urban growth. The growing demand for residential housing in Zimbabwe has boosted the demand for timber supplies, while regional demand has been continuously expanding over the years. In general, securing a sustainable supply of raw materials for pyrolysis is made feasible by the continuous annual output of pine sawdust.

Pine sawdust was often thought to have little economic value by millers and was frequently burned in open fields or dumped in the ground, polluting the environment [6]. Researchers are investigating various methods of utilizing waste biomass as fuel as a result of the rise in demand for waste-to-energy usage. Most experts agree that biomass pyrolysis is a competent way to produce bio-energy from a range of wastes [7]. This method increases the effectiveness of waste management and has great potential for generating renewable and low-carbon energy [8].

Among the thermochemical conversion processes, pyrolysis, which is the breakdown of organic material without the presence of oxygen, is a potential method for utilizing waste biomass. It has been extensively utilized to transform biomass into gaseous, liquid (tar or oil), and solid (char) fuels [9]. The product composition is often related to the pyrolysis operating conditions. Low process temperatures and long vapour residence periods promote char creation, high process temperatures and long vapour residence periods promote the conversion of biomass to gas, while intermediate process temperatures and short vapour residence periods are ideal for liquid production [10]. Due to its high energy content, the solid product is mainly used either directly as an energy source in boilers, or as a feedstock to create activated carbon. Even though the generated gas is a by-product, its main constituents are carbon monoxide and methane, which makes it useable as fuel when burnt. The liquid product known as tars may be added to the feedstock used in petroleum refineries, improved by catalysts to generate premium-grade refined fuels, or it may have potential use as a chemical feedstock.

The simulation of biomass pyrolysis has made use of a variety of commercially available computer-based modelling and simulation programs, including ChemCAD, Fluent, Aspen HYSYS®, and Aspen Plus®. Aspen Plus® is the most popular of these programs for biomass pyrolysis simulations because it comes with a built-in library model for determining the solid properties. As a result, it is more adept at identifying solid components than other applications. Additionally, FORTRAN code, an imperative programming language, is used in conjunction with Aspen Plus® to facilitate the creation of modifications and numerical computations.

Aspen Plus® simulator has been used to model the biomass pyrolysis process for the generation of bio-oil in several in-depth investigations. For instance, Liu et al. [11] simulated the thermochemical conversion of sugarcane bagasse and rice straw. The influence of pyrolysis temperature on product yield was examined by changing the temperature from 300 to 800 °C. Rosha et al. [12] simulated the pyrolysis of biomass using Aspen Plus® for the production of renewable fuel. The authors carried out a sensitivity analysis to find out the optimum operating conditions by varying the operation temperature, feed residence time and reactor volume. Xianjun et al. [13] modelled the pyrolysis of rice husk using Aspen Plus® to determine the non-condensable gas (NCG) yield and properties obtained by varying temperatures between 350 and 600 °C.

The method that has been widely researched is heating biomass with an external heat source. The benefit of pyrolysis is that the end-product yield may be changed depending on the operating conditions; such as process temperature and heating rate [14]. In recent years, MAP has emerged as a viable alternative to conventional pyrolysis, primarily due to its fast-heating rate, selective heating, volumetric heating, and uniform heating, which speed up reaction rates and boost energy efficiency. Instant on/off control made possible by microwave heating makes operation simple and improves product quality and yield. Additionally, it limits pollution emissions and decreases the production of hazardous products, making the process environmentally beneficial [15]. According to research by Yu et al. [16] on the interactions between microwaves and starch, microwave-assisted hydrolysis uses 47% less energy than traditional conductive heating to produce equivalent product yields. Li et al. [17] performed pyrolysis studies in the presence and absence of microwave irradiation in a fixed-bed pyrolysis reactor. The outcomes demonstrated that microwave heating enhanced the liquid product yield and composition, which merits further exploration in the future. Lam et al. [18] observed that microwave pyrolysis may be economically feasible for processing used cooking oil and waste plastics. It also showed potential as a viable method for bio-energy production, offering better process characteristics and effective synthesis of renewable liquid fuels.

The majority of past research has reported the effects of certain parameters while holding other process variables constant at stated levels. This method fails to capture the overall impact of all process factors. Finding the ideal amounts takes time and needs several tests, some of which may be inaccurate. By jointly optimizing all the process parameters using statistical experimental design, such as RSM, these drawbacks of a traditional method may be overcome [19]. The RSM is an effective instrument for investigating the relationships between two or more factors. It primarily consists of a specific combination of mathematical and statistical methods for experimental planning, model creation, analyzing the impacts of variables, and looking for the best combinations of variables to predict certain outcomes [20].

This study aims at optimizing the process conditions to extract high yields of the desired product from used pine sawdust. A reliable numerical simulation model for the MAP of pine sawdust was created. To understand the connection between reaction temperature and pressure and product yield, and to come up with the optimal operating conditions for producing the highest amount of bio-oil from pine sawdust by MAP, simulations were carried out by RSM based on CCD. This may therefore be used to forecast the viability of producing bio-oil from low-cost biomass sources like pine sawdust using MAP, increasing the effectiveness of its utilization.

## 2 Materials and methods

### 2.1 Experimental data

The pine sawdust samples employed in this study were obtained at selected sawmills processing the same wood species in the eastern region of the country. The feedstock was oven dried at 110 °C and sieved through ISO Retsch test sieves of 2 mm mesh size according to ASTM D 410-84. This step was necessary to remove oversized wood chips in the pine sawdust to achieve a uniform particle size distribution. The proximate analysis was carried out according to modified procedures (ASTM D 3173, ASTM D3175 and ASTM D 3174). The ultimate analysis was carried out in accordance with ASTM D5373 (2014) and a Thermo Scientific™ FLASH 2000 CHNS/O Analyzer was employed in the analysis. In the experimental runs, 100 g of pine sawdust was mixed with 10 g of microwave-absorbent activated carbon. The microwave reactor used has a cavity volume of 42 L, a rated microwave output of 950 Watts and an operating frequency of 2450 MHz. Helium at a flowrate of 3 LPM served as carrier gas and to create an inert environment in the reactor. The samples were subjected to microwave heating at maximum power for 30 min. A very short residence time of ∼2 s was maintained to control the further conversion of large molecules in bio-oil into small molecules which are more stable under a thermodynamic equilibrium state. The volatiles that evolved during the pyrolysis process were forced to pass through a condensing system utilizing water as a coolant. When the vapours go through the cooling lines the condensable compounds condense from the gaseous vapour forming bio-oil which is collected in a collection flask. Both the reactor and the condensing system were weighed before and after the pyrolysis experiments to measure the liquid and solid yields. The gas yield was determined by difference.

### 2.2 Simulation model

Aspen Plus® V11 software was utilized for the modelling and simulation of the pyrolysis of pine sawdust. It has built-in tools to enable the calculation of process energy and mass balances, reaction kinetics, chemical equilibrium and process optimization. Complicated and comprehensive systems can be modelled from the extensive databases, physical properties and thermodynamic models in the software. The ability to handle conventional and non-conventional solids, liquids and gaseous compounds makes it ideal for modelling chemical processes [21]. There are two types of components in Aspen Plus®: conventional and non-conventional. Conventional components have recognized molecular structures and may be found in several Aspen Plus® databanks. The representation of non-conventional components is based on proximate and ultimate analysis as they lack a molecular formula [22].

#### 2.2.1 Model component specification

The simulation contains mixed streams of conventional and non-conventional solids, liquids and gaseous compounds; therefore, the global stream class is set to MIXCINC. A particle size distribution of 250 μm-14mm was modelled for the pine sawdust samples [23]. For non-conventional components, only enthalpy and density are estimated; these variables are determined by empirical correlations. The HCOALGEN and DCOALIGT methods, which depend on ultimate and proximate analyses, respectively, were selected as specified property methods for enthalpy and density for pine sawdust. Proximate and ultimate analyses are needed to model non-conventional components and the data required to model the pine sawdust in Aspen Plus® is presented in Table 1.

**Table 1:** Pine sawdust composition (wt.%, dry basis).

Proximate analysis	
Moisture Content	7.29
Volatile Matter	78.19
Ash Content	0.28
Fixed Carbon	14.24
Ultimate analysis	
C	51.60
H	5.20
N	0.04
O	43.16^a^

a Calculated from difference.

The conventional and non-conventional components added to the simulation model are presented in Table 2. H_2_O was included in the components specification to accommodate for the feedstock’s moisture content. Since pine sawdust is a non-conventional component, it is decomposed to lignin, hemicellulose and cellulose species during the simulation and modelling process. Cellulose is denoted by its monomer C_6_H_10_O_5_ [24] and hemicellulose is represented by glucomannan and xylan monomers (C_5_H_8_O_4_). Lignin is a complex organic polymer made up of phenolic monomers. Tannin (C_15_H_12_O_7_), C-rich lignin (C_15_H_14_O_4_), O-rich lignin (C_20_H_22_O_10_) and H-rich lignin (C_22_H_28_O_9_) are used as monomers for lignin. The pyrolysis intermediate products and end products modelled in the simulation are listed in Table 2.

**Table 2:** Model component specifications.

Component ID	Type	Component	Molecular Formula
C	Solid	Carbon graphite	C
TANN	Solid	Tannin	C_15_H_12_O_7_
METHY-01	Solid	Carbon-rich lignin	C_15_H_14_O_4_S
LIGO	Solid	Oxygen-rich lignin	C_20_H_22_O_10_
LIGH	Solid	Hydrogen-rich lignin	C_22_H_28_O_9_
GMSW	Solid	Hemicellulose-glucomannan	C_5_H_8_O_4_
XYHW	Solid	Hemicellulose- xylan	C_5_H_8_O_4_
CELL	Solid	Cellulose	C_6_H_10_O_5_
ASH	Solid	Calcium oxide	CaO
LIG	Solid	Secondary lignin intermediate	C_11_H_12_O_4_
METHY-02	Solid	Carbon-rich lignin intermediate	C_15_H_14_O_4_S
LIGOH	Solid	Hydrogen/Oxygen-rich lignin intermediate	C_19_H_22_O_8_
HCE1	Solid	Activated hemicellulose 1	C_5_H_8_O_4_
HCE2	Solid	Activated hemicellulose 2	C_5_H_8_O_4_
CELLA	Solid	Activated cellulose	C_6_H_10_O_5_
ITANN	Solid	Tannin intermediate	C_8_H_4_O_4_
CHAR	Solid	Carbon graphite	C
HMWL	Solid	High-molecular weight lignin	C_24_H_28_O_4_
CH4	Conventional	Methane	CH_4_
H2	Conventional	Hydrogen	H2
CO2	Conventional	Carbon dioxide	CO_2_
CO	Conventional	Carbon monoxide	CO
HE	Conventional	Helium	He
H2O	Conventional	Water	H_2_O
H2S	Conventional	Hydrogen sulphide	H_2_S
O2	Conventional	Oxygen	O_2_
CH3OH	Conventional	Methanol	CH_4_O
N2	Conventional	Nitrogen	N_2_
HCOOH	Conventional	Formic acid	CH_2_O_2_
CH2O	Conventional	Formaldehyde	CH_2_O
COUMARYL	Conventional	Ethyl-benzoate	C_9_H_10_O_2_
ANISOLE	Conventional	Methyl-phenyl-ether	C_7_H_8_O
HMFU	Conventional	1,2,3-benzenetriol	C_6_H_6_O_3_
PHENOL	Conventional	Phenol	C_6_H_6_O
LVG	Conventional	Levoglucosan	C_6_H_10_O_5_
XYLAN	Conventional	Xylosan	C_5_H_8_O_4_
FURF	Conventional	Furfural	C_5_H_4_O_2_
C3H6O2	Conventional	1,3-dioxolane	C_3_H_6_O_2_
ALD3	Conventional	Propylene-oxide	C_3_H_6_O
ACROL	Conventional	Acrolein	C_3_H_4_O
C2H5OH	Conventional	Ethanol	C_2_H_6_O
GLYCO-01	Conventional	Glycol-aldehyde	C_2_H_4_O_2_
ACETI-01	Conventional	Acetic-acid	C_2_H_4_O_2_
CH3CHO	Conventional	Acetaldehyde	C_2_H_4_O
C2H4	Conventional	Ethylene	C_2_H_4_
GLYOX	Conventional	Glyoxal	C_2_H_2_O_2_
FFA	Conventional	Cyclotetradecane-1,8-dione-ethylene-ketal	C_18_H_32_O_4_
FE2MACR	Conventional	Sinapyl aldehyde	C_11_H_12_O_4_
BIOMASS	Non-conventional		
DRY-BIOM	Non-conventional		

The Peng-Robinson-Boston-Mathias (PR-BM) method was chosen as the property’s technique for the simulation. This property method is employed when the model consists of hydrocarbons and light gases [25]. When temperatures are extremely high, the temperature-dependent parameter alpha enhances the correlation of pure component vapour pressure [26]. All solid components must have values for their molecular weight, solid molar heat capacity, solid molar volume and standard solid heat of formation model parameters to employ the PR-BM technique. These values are presented in Table 3. All conventional fluid components must have values for their molecular weight, ideal gas standard state heat of formation, critical pressure and temperature, vapour pressure, acentric factor, and ideal gas molar heat capacity characteristics. These values are presented in Table 4.

**Table 3:** Estimated solid property model parameters for conventional solids [27].

Component name	Molecular weight, kg/kmol	Standard solid enthalpy of formation, kJ/kmol	Heat capacity model coefficients		Solid density, kmol/m^3^
			C_1_, kJ/kmol-K	C_2_, kJ/kmol-K^2^	
Tannin and lignin					
Secondary lignin intermediate	208.21388	−729,310	13.2251	0.82834	7.3002
Carbon-rich lignin	258.27376	−759,390	16.4048	1.02749	5.8852
Carbon-rich lignin intermediate	258.27376	−759,390	16.4048	1.02749	5.8852
Hydrogen-rich lignin	436.45892	−1722,700	27.7226	1.73636	3.4826
Oxygen-rich lignin	422.38868	−1847,500	26.8289	1.68039	3.5986
Hydrogen/Oxygen-rich lignin intermediate	378.37888	−1429,200	24.0335	1.50530	4.0171
High-molecular weight lignin	380.48392	−958,260	24.1672	1.51386	3.9949
Tannin intermediate	164.11736	−616,980	10.4242	0.65291	9.2617
Tannin	304.25608	−1079,700	19.3254	1.21042	4.9958
Cellulose species					
Cellulose	162.1424	−1019,000	−1.5328	0.67527	9.3745
Activated cellulose	162.1424	−1019,000	−1.5328	0.67527	9.3745
Hemicellulose species					
Hemicellulose- xylan	132.11612	−759,200	−1.2489	0.55022	11.5050
Hemicellulose-glucomannan	132.11612	−759,200	−1.2489	0.55022	11.5050
Activated hemicellulose 1	132.11612	−759,200	−1.2489	0.55022	11.5050
Activated hemicellulose 2	132.11612	−759,200	−1.2489	0.55022	11.5050

**Table 4:** Estimated fluid property model parameters for conventional fluids [27].

	1,3-dioxolane	Ethyl-benzoate	Sinapyl aldehyde	Xylosan
Molecular weight, kg/kmol	74.07944	150.1772	208.21388	132.11612
Ideal gas enthalpy of formation, kJ/kmol	−345,300	−193,500	−483,800	−642,300
Critical temperature, K	605.0	791.4	837.9	744.3
Critical pressure, bar	56.36	56.90	29.25	2.134
Acentric factor	1.133	1.198	0.981	0.292
Ideal gas heat capacity estimates, J/mol-K:				
Cp*^,ig^ (298 K)	91.46	177.30	240.85	142.76
Cp*^,ig^ (400 K)	109.33	225.11	302.37	179.06
Cp*^,ig^ (500 K)	126.37	264.73	357.37	215.39
Cp*^,ig^ (600 K)	141.02	296.16	402.54	240.55
Cp*^,ig^ (800 K)	164.75	342.81	471.36	281.03
Cp*^,ig^ (1000 K)	182.00	374.75	520.31	307.48
Aly-Lee Cp*^,ig^ equation coefficients:				
C_CP,1_, J/kmol-K	77793.84	128972.6	190226.6	115298.4
C_CP,2_, J/kmol-K	106997.7	342667.4	491979.1	224458.5
C_CP,3_, K	814.165	1575.222	1728.691	824.2086
C_CP,4_, J/kmol-K	66750.56	266861.9	371592.3	59411.96
C_CP,5_, K	2048.402	728.2816	797.2112	2302.592
C_CP,6_, K	298	298	298	298
C_CP,7_, K	1000	1000	1000	1000
Extended Antoine equation coefficients, K/Pa:				
C_PL_,_1_	136.9781	286.7075	286.6149	135.2637
C_PL,2_	−13924.84	−25124.63	−25391.53	−14336.53
C_PL,3_	−15.46495	−37.26739	−37.28766	−15.74501
C_PL,4_	1.303768e-17	1.48627e-5	1.36118e-5	2.245921e-18
C_PL,5_	6	2	2	6
C_PL,6_	261.15	406.15	406.15	455.4
C_PL,7_	605	791.4	837.9	744.3

#### 2.2.2 Reactor model description

Three reactor blocks, the RSTOIC, RYIELD, and RGIBBS reactors, were used in conjunction to simulate the pyrolysis process. Stoichiometric equations are used in the RSTOIC block, a stoichiometric reactor. This served as a model for the drying of pine sawdust before heating. The conversion of pine sawdust to conventional simulation components takes place in the RYIELD reactor. By minimizing Gibbs free energy, the RGIBBS reactor determines the product components' distribution and phase equilibrium. The chemical equilibrium may be expressed as Eq. (1) for a system operating at constant temperature and pressure:

(1) $$dG=\sum_{i=1}^{k}\mu_{i}n_{i}{dn}_{i}$$

where *k* is the sum of chemical species present in the reaction, *G* is the Gibbs free energy, $\mu_{i}$ is the chemical potential of species *i* and *n*_*i*_ denotes the number of moles of species *i*. The goal is to identify the values of *n*_*i*_ that will reduce the value of the Gibbs free energy. Aspen Plus® employs a non-stoichiometric method to determine the solution. If the mass balance is assumed, the expression, known as the objective function, can be expressed as Eq. (2).

(2) $$G=\sum_{i=1}^{k}n_{i}\Delta G_{i}^{{}^{\circ}}+RT\sum_{i=1}^{k}n_{i}Iny_{i}+RT\sum_{i=1}^{k}n_{i}InP$$

where *T* denotes the temperature, *P* denotes the pressure, *y*_*i*_ denotes the mole fraction and $G_{i}^{{}^{\circ}}$ denotes the standard Gibbs free energy of formation. This serves as the foundation for the computations carried out by the software to find thermodynamically viable results. Stoichiometric equations are not necessary for the Gibbs reactor or the yield reactor to be specified. The descriptions of each unit used in the simulation are listed in Table 5. The assumptions that were considered in developing the model are:

• The Aspen Plus® pyrolysis model is a steady-state, isothermal model that makes use of sequential-modular computing. Time-dependent factors like heating rate and residence time cannot be directly investigated since the model is not transient or dynamic.
• 95% of the moisture in the biomass is evaporated during the drying process.
• Biochar produced is assumed to be made up of elemental carbon.
• All components except for helium, which is regarded as inert, participate in the chemical process.

**Table 5:** Model block description.

Aspen Plus® ID	Block ID	Description
RSTOIC	DRYER	Models the removal of moisture from the raw pine sawdust.
SEP	SEP-1	Models the separation of water vapour and dry pine sawdust.
MIXER	MIXER-01/02	Merges several streams to form one stream.
CRUSHER	CRUSHER	Models the particle size reduction of dried biomass.
SCREEN	SCREEN	Utilized to remove oversized biomass particles.
RYIELD	DECOMP	Models the decomposition of pine sawdust to conventional components.
RGIBBS	PYROLY	Determines the distribution of the pyrolysis products using the Gibbs free energy minimization approach.
CYCLONE	CYCLONE	Separates the biochar from the gaseous stream.
HEATX	HEX	Lowers the vapour stream temperature, causing the pyrolysis liquid products to condense.
FLASH2	SEP-2	Separates the condensable and non-condensable fractions of the pyrolysis products.

#### 2.2.3 Description of the process model

**Fig. 1:**
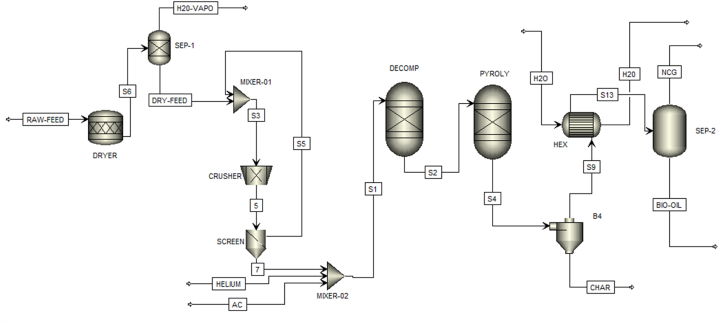
Aspen Plus® simulation flow diagram for the conversion of pine sawdust to pyrolysis products.

The process flow chart developed in Aspen Plus® is demonstrated in Fig. 1 and the stream information is presented in Table 6. Raw biomass at 200 kg/h (25 °C and 1 atm) enters the RSTOIC reactor and is dried at 125 °C to remove moisture in the biomass. A biomass fractional conversion of 1 has been specified [11,28] and the drying of biomass was modelled using the stochiometric equation in Eq. (3). The separator block is used to remove the water vapour that evolves during the drying process. A crusher was modelled to reduce the biomass particle size to 2 mm and the screen separates the oversized biomass particles which are then recycled into the crusher. The pine sawdust is transformed into conventional simulation components by the RYIELD reactor at 500 °C and 1 atm. Helium gas at 16 kmol/h is added to the stream to maintain an inert environment in the pyrolysis reactors. Activated carbon acts as a microwave absorbent when pyrolysis is taking place. The yield fractions of pine sawdust are specified based on research by Caudle et al. [29] and the decomposition fractions are presented in Table 7. The RGIBBS reactor estimates the distribution of the products by minimizing Gibbs free energy. Helium gas was defined as an inert component in the RGIBBS reactor. The operating pressure and temperature of the RGIBBS reactor was specified at 1 bar and 500 °C respectively. The relationship between the bio-oil output and the operating temperature was examined by varying the temperatures of the RYIELD reactor. The solid fraction (char) was separated from the product stream with the aid of a cyclone. The rest of the product stream is condensed to ambient temperature to separate the gaseous products from the liquid fraction (bio-oil).

(3) $$BIOMASS\;\to \;0.95\;DRY\;BIOMASS+0.0027754\;H_{2}O$$

**Table 6:** Aspen Plus® model stream information.

Stream ID	Description
RAW-FEED	Raw biomass feed (pine sawdust) enters the RSTOIC block for drying at 25 °C and 1 bar.
S6	A mixture of dried pine sawdust and moisture.
H2O-VAPO	The stream consists of moisture separated from the pine sawdust.
DRY-FEED	The stream consists of dried biomass feed.
S5	Particles >2 mm are recycled to the crusher.
HELIUM	The stream consists of helium gas at 16 kmol/h. Helium served a carrier gas and provided an inert environment in the pyrolysis reactors.
AC	The stream consists of activated carbon which acts as a microwave absorbent.
S2	The stream consists of decomposed biomass components from the RYIELD reactor.
S4	The stream consists of the pyrolysis products from the RGIBBS reactor.
S9	A mixture of the non-condensable and condensable vapour fractions.
CHAR	The stream consists of the by-product of pyrolysis, char.
H2O	Cooling water is used in the heat exchanger.
S13	A mixture of the condensed fraction and the non-condensable fraction of the pyrolysis products from the heat exchanger.
NCG	The stream is composed of non-condensable gases.
BIO-OIL	The stream constitutes the condensable fraction of the pyrolysis products, bio-oil.

**Table 7:** Biomass decomposition yield fractions.

Component	Basis	Yield
CELL	Mass	0.4385
GMSW	Mass	0.2191
METHY-01	Mass	0.0471
LIGH	Mass	0.1199
LIGO	Mass	0.1084
TANN	Mass	0.0125
ASH	Mass	0.0046
XYHW	Mass	0.0499

#### 2.2.4 Process sensitivity analysis

The process sensitivity analysis was performed using the developed simulation model by adjusting reactor pressure from 1 to 3.3 bar and temperature from 340 to 700 °C. The distribution of the product was then examined in relation to temperature and pressure.

#### 2.2.5 Response surface methodology

The main objective of this analysis was to come up a regression model that would evaluate system performance by taking into account how the key parameters interacted. Design-Expert® Software was used to develop a design matrix using the Aspen Plus® simulated data. The parameters for biomass fast pyrolysis were optimized using RSM based on CCD. A second-order model has been fitted using the CCD, a standard RSM design. CCD is an excellent approach for fitting a quadratic surface and aids in minimizing the number of tests required to maximize the effective parameters and examine how the parameters interact.

Analysis of variance (ANOVA) was evaluated using statistical analysis of the model. ANOVA enables determining the significance of the effect and interaction of the examined parameters in relation to the experimental error. To evaluate the significance of the effects, statistical F-tests were utilized. It is applied to compare statistical models that have been fitted to data sets to determine which model better represents the population that the data were sampled. By doing an ANOVA at a 95% confidence level and assessing the model’s quality, the objective response regression models were developed. According to the second-order quadratic equation shown in Eq. (4), the response variable interaction was established [30].

(4) $$y=\beta_{0}+\sum_{i=1}^{n}\beta_{i}x_{i}+\sum_{i=1}^{n}\beta_{ii}x_{i}^{2}+\sum_{i=1}^{n-1}\sum_{j=1}^{n}\beta_{ij}x_{i}x_{j}+\varepsilon$$

where *y* denotes the output response, *x* denotes the decision parameter, $\beta_{i}$ denotes the coefficient, *n* denotes the total sum of the variables, and ε is the statistical error.

The constructed regression model’s accuracy was measured using the regression coefficient ($R^{2}$) and the adjusted regression coefficient ($R_{adj}^{2}$) values. These variables were determined using Eq. (5) [30]:

(5) $$R_{adj}^{2}=1-\left[\frac{\left(\frac{{SS}_{R}}{n-p}\right)}{\left(\frac{{SS}_{T}}{n-1}\right)}\right]=1-\frac{\left(1-R^{2}\right)\left(n-1\right)}{1-p}$$

Where ${SS}_{R}$ and ${SS}_{T}$ are determined using Eqs. (6), (7)).

(6) $${SS}_{R}=\sum_{i=1}^{n}{\left(y_{i}-y_{j}\right)}^{2}$$

(7) $${SS}_{T}=\sum_{i=1}^{n}y_{i}^{2}-\frac{\left(\sum_{i=1}^{n}y_{i}^{2}\right)}{n}$$

where *y*_*i*_ and *y*_*j*_, respectively, denote the observations and the fitted observations.

The p-value, a key parameter in the model, is usually perceived as insignificant if its value surpasses 0.05 [31]. The degree of fit is measured by R^2^, which was calculated using Eq. (8) [30].

(8) $$R^{2}=1-\frac{{SS}_{R}}{{SS}_{T}}$$

The values of $R^{2}$ and $R_{adj}^{2}$ were between 0 and 100%. A value of more than 90% means the model is accurate [30,32]. Further evidence for a good model is provided by the less than 0.2 difference between $R^{2}$ and $R_{adj}^{2}$ [32].

#### 2.2.6 Model validation

Model validation was performed using the product distribution results attained from the experimental setup described in section 2.1. A set of three published experimental results acquired under circumstances comparable to the simulations' operating limitations were used to further validate the simulation results. The initial validation was performed using Ningbo et al. (2015) experimental findings [33]. In their experimental work, fast pyrolysis of pine wood was performed in a screw reactor to examine the influence of solid residence time and pyrolysis temperature on the distribution of products and energy. Secondly, the comparison employed DeSisto et al. (2010) experimental findings [34]. In their study, pine sawdust was pyrolyzed between 400 and 600 °C in a fluidized-bed pyrolysis reactor. Lastly, the comparison employed Zhang et al. (2017) experimental findings [35]. The study employed fluidized-bed pyrolysis to carry out the pyrolysis of pine wood.

The deviation of the simulation results from the experimental results from the literature was measured using the root mean square error (RMSE). This was established using Equation (9).

(9) $$RMSE=\sqrt{\sum {\left(X_{s}-X_{e}\right)}^{2}/N}$$

where X_s_ is the simulated result, X_e_ is the experimental result and N is the number of data sets.

### 2.3 Economic evaluation

The Aspen Plus Economic Analyzer was employed in determining the equipment costs. Scaling and installation factors were used to adapt the price of the purchased machinery to the required size, operating pressure, and building materials. Using a Chemical Engineering Plant Cost Index (CEPCI) of 699.0, installed equipment costs were then updated for the year 2022. Peters and Timmerhaus' approach was employed in the determination of the total project investment (TPI), as presented in Table 8 [36,37]. The MSP of bio-oil, described as the minimum market price which provides a net present value (NPV) equal to zero at a set Internal Rate of Return (IRR); 10% nominal or 22% desired, was determined in this study using a discounted cash flow rate of return (DCFROR) analysis. Table 9 lists the assumptions used in the DCFROR analysis. A 25% change in an economic parameter’s impact on the MSP of bio-oil was taken into account while conducting economic and process sensitivity analysis to determine which economic parameters provide the most investment risk.

**Table 8:** TPI estimation method [36,37].

Parameter	Factor
Total purchased equipment cost (TPEC)	TPEC
TIC	3.02*TPEC
Indirect cost (IC)	0.89*TPEC
Total direct and indirect cost (TDIC)	TIC + IC
Contingency	0.2* TDIC
Fixed capital investment (FCI)	TDIC + contingency
Location factor (LF)	0.1*FCI
**Total project investment (TPI)**	**FCI + LF**

**Table 9:** DCFROR assumptions.

Parameter	Assumption
Plant life	25 years
Equity	40%
Loan interest	7.5%
Loan term	10 years
Income tax rate	39%

## 3 Results and discussion

### 3.1 Validity of the model

Based on data from MAP, simulations for the fast pyrolysis of pine sawdust were performed in Aspen Plus® software. The experimental results and the output of bio-oil, biochar, and NCGs were compared to assess the validity of the proposed microwave-assisted fast pyrolysis model. Using input and output variables including reactor temperature, biomass properties, and absorbent and inert gas flow rates, among others, the fast pyrolysis process was simulated under similar conditions described in the experimental setup.

**Fig. 2:**
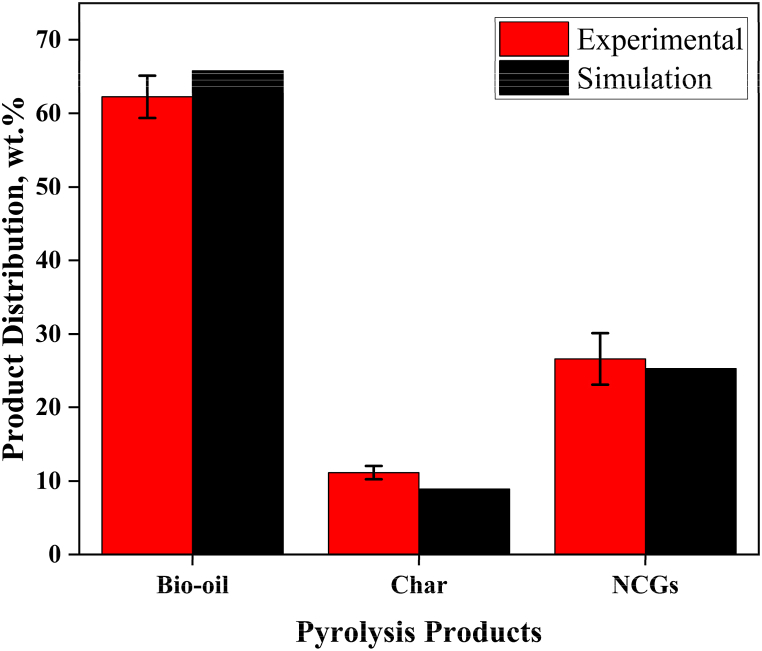
Comparison between experimental findings and Aspen Plus® model results.

The simulation was carried out at 500 °C using Aspen Plus® to estimate the output of bio-oil, biochar, and NCGs. This simulation was run after specifying the physical and chemical parameters of each component utilized in the proposed model. Fig. 2 compares experimental data with the product yield results that were obtained. The RMSE illustrates how closely simulated results match experimental data. The product distribution of the simulated model and the experimental results matched satisfactorily with an RMSE of 3.55%, 2.24% and 1.31% for bio-oil, char and NCGs respectively.

**Fig. 3:**
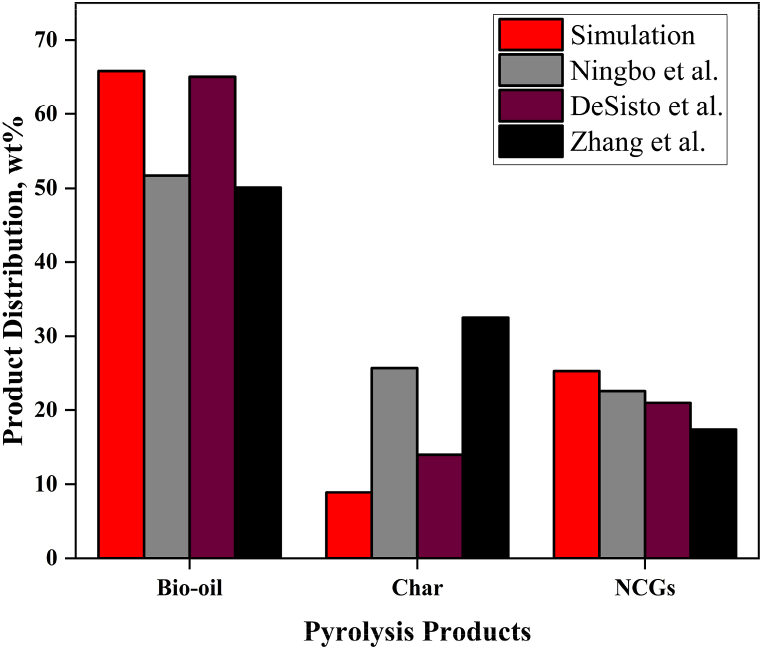
Comparison between published works and Aspen Plus® model results.

In addition, the model was compared with three sets of published experimental results as shown in Fig. 3. The model product distribution was fairly consistent with the experimental works of DeSisto et al. with an RMSE of 0.8% for bio-oil [34]. However, lower bio-oil outputs of 51.7 wt% and 50.1 wt% were obtained from the experimental works of Ningbo et al. [33] and Zhang et al. [35] respectively. The lower bio-oil yields attribute to higher temperatures (600 °C) employed during the pyrolysis process. At higher pyrolysis temperatures, the long-chain macromolecules are broken down into smaller fragments and the pyrolysis vapours undergo secondary cracking. Lower char yields may be the result of a more extensive primary breakdown of biomass and subsequent decomposition of the solid product at higher temperatures [33]. Both Lu et al. [38] and Heidari et al. [39] observed comparable trends in the product distribution temperatures between 500 and 800 °C.

### 3.2 Effects of process parameters on product distribution

**Fig. 4:**
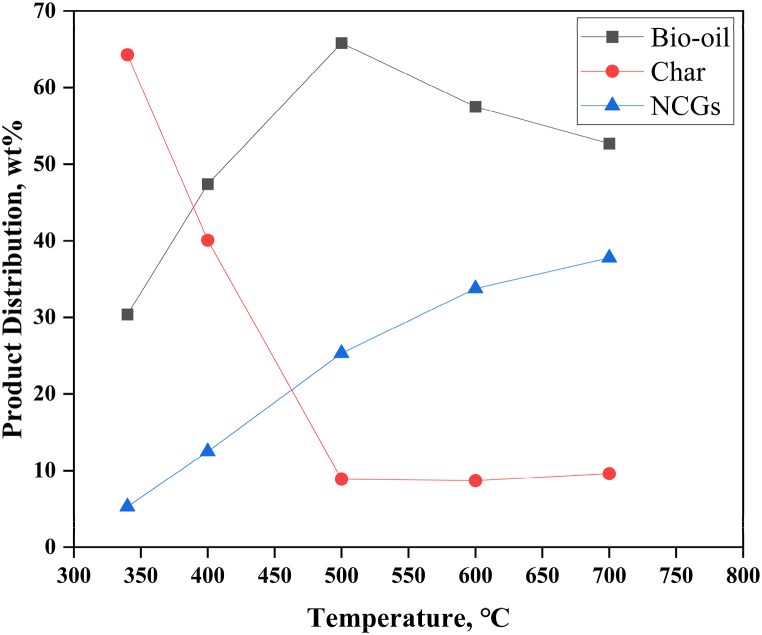
Effect of temperature on pyrolysis product output.

Table 10 shows the pyrolysis product distribution at various pyrolysis temperatures and pressures. The influence of pyrolysis temperature on the product distribution is illustrated in Fig. 4. The production of char was favoured at low pyrolysis temperatures because lower temperatures tend to favour charring processes that produce coke. The bio-oil product yield increases with increasing pyrolysis temperature, but only until a certain point, at which point it begins to fall [40]. At temperatures between 500 and 550 °C, the bio-oil product yield reached its maximum; as temperatures are further increased, the yield gradually declined. The observed decrease in bio-oil production was attributed to subsequent thermal cracking reactions between pyrolysis vapours and tar fragments to produce true vapours, which enhanced the yield of NCGs. In addition, regardless of the heating technique utilized, Binti Mohd found that the formation of NCGs often rises as the pyrolysis temperature rises [41]. Thus, at higher temperatures, both tar and char are transformed into lighter hydrocarbons like syngas. The bio-oil product yield has regularly been observed to improve initially with temperature increase up to a particular temperature, where the production would decrease as temperatures are further increased. Additionally, comparable results were obtained regarding the effect of temperature on the rapid pyrolysis of biomass feedstock in a fluidized-bed reactor by Lu et al. [38] and Heidari et al. [39].

**Table 10:** Distribution of pyrolysis products at various pyrolysis temperatures and pressures.

Variable modified	Process inputs		Product distribution (wt.%)		
	Temperature (°C)	Pressure (atm)	Bio-oil	Char	NCGs
Baseline	500	1	65.8	8.9	25.3
Reactor Temperature	340	1	30.4	64.3	5.3
	400	1	47.4	40.1	12.5
	600	1	57.5	8.7	33.8
	700	1	52.7	9.6	37.8
Reactor Pressure	500	1.3	60.1	19.0	20.9
	500	1.8	53.7	11.1	25.3
	500	2.8	64.9	8.3	26.9
	500	3.3	63.6	8.1	28.3

**Fig. 5:**
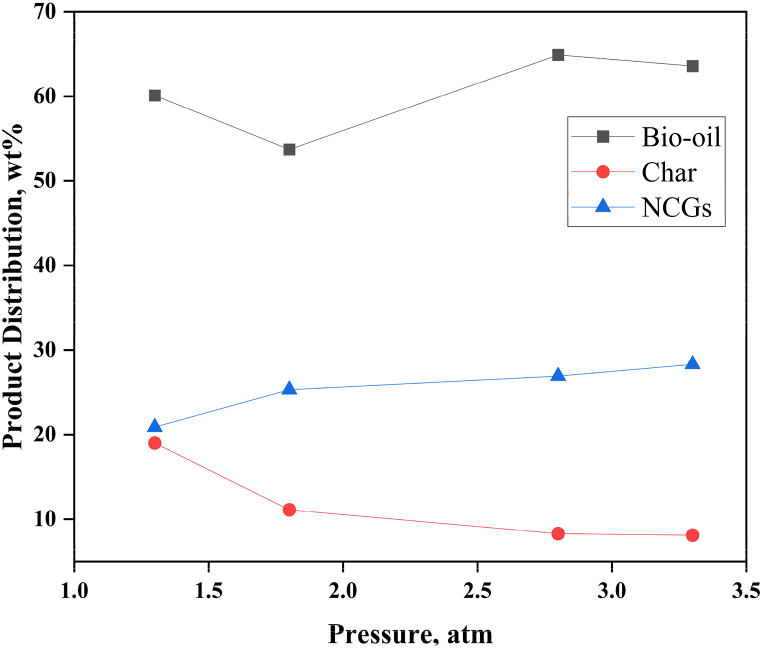
Effect of reactor pressure on pyrolysis product output.

The influence of reactor pressure on product distribution was also investigated. The effects of reactor pressure on product distribution are shown in Fig. 5. The reactor pressure has no significant effect on pyrolysis product distribution. However, because of its inverse relationship to the volumetric density of the vapour phase, it indirectly affects the reactor’s residence time [29].

### 3.3 Regression models and ANOVA

In terms of the coded factors with significant variables, the final model regression equations for bio-oil, char, and NCGs yield are shown in Eqs. (10), (11), (12)), respectively.

(10) $$Bio-oil\;Yield\;\left(\%\right)=-133.27464+\left(0.671978A\right)+4.16109B-3.99303^{-16}AB-0.000579A^{2}-0.967696B^{2}$$

(11) $$Char\;Yield\left(\%\right)=291.49722-0.907127A-5.1692B+7.85616^{-16}AB+0.00072A^{2}+1.20214B^{2}$$

(12) $$NCGs\;Yield\;\left(\%\right)=-51.22833+0.208476A+1.74404B-1.50616^{-16}AB-0.000119A^{2}-0.405592B^{2}$$

Where A is the temperature and B is the pressure.

Table 11 presents the results of the ANOVA for the output of bio-oil, char, and NCGs. The significance of the models is demonstrated by the low p-values (less than 0.0001) and high F-values (74.94, 156.55, and 174.52, respectively) for the bio-oil, char, and NCGs yield regression models. A high F-value indicates that the model is significant and there is only a 0.01% likelihood that F-values this big are caused by noise. The linear and square terms are significant, based on the ANOVA results. However, the effect of reactor pressure on the output of bio-oil insignificant. The correlation coefficient (R^2^) measures how much the independent factor variables in the model’s response have been able to reduce the response’s variability. R^2^ = 0 denotes that the fit does not predict the response any better than the mean response as a whole, and R^2^ = 1 denotes that the fit is perfect (the errors are all zero). Regression models for the product distribution of bio-oil, char, and NCGs had R^2^ values of 98.17%, 99.11%, and 99.20%, respectively. The high R^2^ values indicate that the bio-oil, char, and NCGs yield regression models fit the experimental findings with a level of acceptable precision. Additionally, the adjusted R^2^ values of 96.86%, 98.48%, and 98.64% for the respective yields of bio-oil, char, and NCGs are reasonably close to the corresponding R^2^ values. The difference of less than 0.2 between the Predicted R^2^ values and the Adjusted R^2^ values for all the regression models further implies the significance of the model. These findings indicate that there is very little probability of including an insignificant term in the model. As a result, the response variables may be precisely determined by the regression models.

**Table 11:** ANOVA results for pyrolysis products yield.

Constant	df	Bio-oil Yield				Char Yield				NCGs Yield			
		Sum of Squares	Mean Squares	F-value	p-value	Sum of Squares	Mean Squares	F-value	p-value	Sum of Squares	Mean Squares	F-value	p-value
**Model**	5	3729.33	745.87	74.94	<0.0001	10315.4	2063.08	156.55	<0.0001	1983.77	396.75	174.52	<0.0001
A-Temperature	1	1273.88	1273.88	127.99	<0.0001	6517.19	6517.19	494.53	<0.0001	1881.14	1881.14	827.44	<0.0001
B-Pressure	1	0.0000	0.0000	0.0000	1.0000	0.0000	0.0000	0.0000	1.0000	0.0000	0.0000	0.0000	1.0000
AB	1	0.0000	0.0000	0.0000	1.0000	0.0000	0.0000	0.0000	1.0000	0.0000	0.0000	0.0000	1.0000
A^2^	1	2445.83	2445.83	245.74	<0.0001	3783.28	3783.28	287.08	<0.0001	102.62	102.62	45.14	0.0003
B^2^	1	11.39	11.39	1.14	0.3202	17.58	17.58	1.33	0.2860	2.00	2.00	0.8804	0.3793
**Residual**	7	69.67	9.95			92.25	13.18			15.91	2.27		
Lack of Fit	3	69.67	23.22			92.25	30.75			15.91	5.30		
Pure Error	4	0.0000	0.0000			0.0000	0.0000			0.0000	0.0000		
**Total**	12	3799.00				10407.6				1999.69			
													
***Coefficient of determination***													
R^2^		0.9817				0.9911				0.9920			
Adjusted R^2^		0.9686				0.9848				0.9864			
Predicted R^2^		0.8696				0.9370				0.9434			

**Fig. 6:**
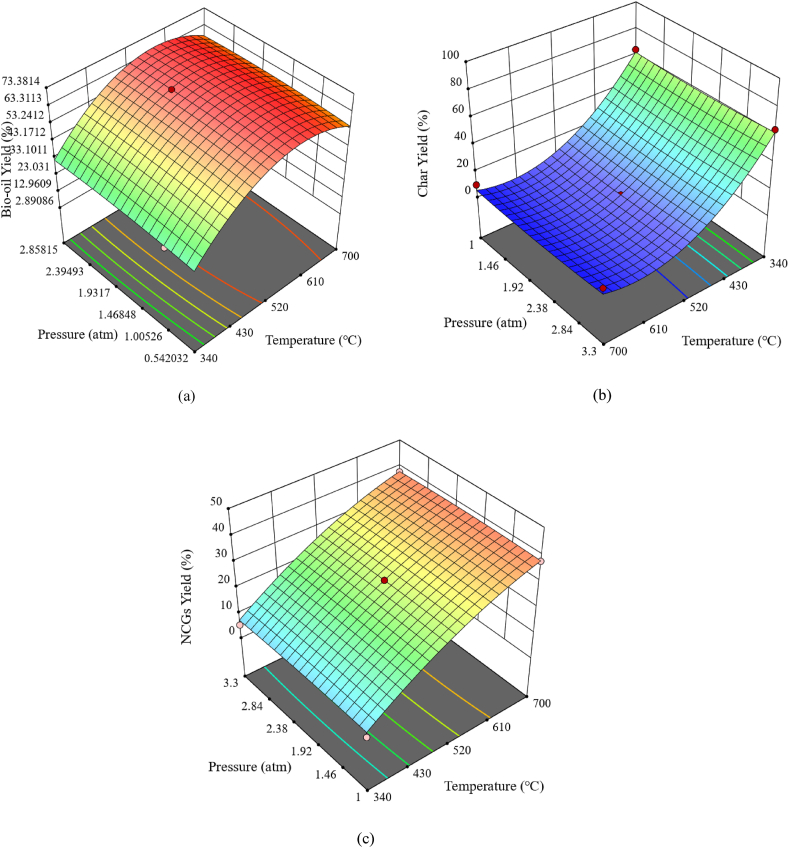
3D response surface plots of the effect of operating temperature and reactor pressure on (a) bio-oil yield, (b) char yield, and (c) NCGs yield.

Three-dimensional (3D) response surface plots of two factors were represented in this study. The purpose of the 3D-surface plot is mainly to classify the surface shape for various parameters used and show the effectiveness of each parameter on the output of bio-oil, char and NCGs [42]. The operating point at which the highest production of pyrolysis products may be achieved is investigated using the synchronized effects of the crucial parameters. Fig. 6 presents the combined effects of reactor temperature and pressure on the production of bio-oil, char, and NCGs. Bio-oil yield increased with increased temperature from 340 to 600 °C and eventually decreased with temperatures exceeding 600 °C. Temperatures between 520 and 600 °C result in a high amount of bio-oil output, with 550 °C and atmospheric pressure as the optimum operating conditions. The effect of reactor pressure bio-oil yield is insignificant, as illustrated in Fig. 6(a). Maximum char yields are obtained at the lowest temperature (340 °C) (Fig. 6(b)) as low temperatures tend to favour the charring process that produces coke. Incomplete or unpyrolyzed biomass might be the result of the increased char output at lower temperatures [34]. It is evident from Fig. 6(c) that the yield of NCGs increases with increasing temperatures. As the NGCs yield increases, the bio-oil yield is observed to decrease with increasing temperature, owing to the secondary cracking reactions of the gaseous and liquid products with rising temperature [39,40]. From the 3D surface plots, the maximum yields of bio-oil were archived at temperatures of 550 °C and atmospheric pressure. Therefore, it can be inferred that reaction temperature has a substantial effect on the conversion of biomass into bio-oil.

However, other parameters such as residence times and heating rate are known to affect the mass yields of the pyrolysis products. The heating rate is crucial in the pyrolysis process since it affects the amount and quality of the end product to some extent. The likelihood of secondary reactions can be eliminated or minimized at low heating rates. A low heating rate also prevents the thermal cracking of biomass, increasing the production of biochar. High heating rates support biomass fragmentation and boost gas and liquid production, which reduces the likelihood of biochar formation [43]. According to Chen et al., enhanced heating rates gave rise to higher mass yields of the liquid product, and the influence of heating rate on biochar being more noticeable at lower temperatures. The mass yield of NCGs is not significantly influenced by the heating rate, but the yield rises with temperature, mainly because of the secondary cracking reactions of the volatiles [44]. Short residence times favour bio-oil production by rapid removal of organic vapours from the reactor, minimizing secondary reactions [45]. A longer residence time gives the biomass components sufficient time to react and promotes repolymerization of the biomass components [43].

### 3.4 Economic evaluation

#### 3.4.1 Capital cost estimation

The findings of the process modelling were utilized to estimate and calculate the cost of the process equipment. The pyrolysis unit contributes between 37.9% to the total installed equipment cost (TIEC). In a study by van Schalkwyk et al. [46], pyrolysis contributes between 39.8 and 44.1% to the TIEC of each biorefinery scenario. Although pyrolysis contributed 31.1% to the total installed equipment cost in the study by Dutta et al. [47], the total installed equipment cost on an annual biorefinery throughput basis was significantly higher than in this study. Due to economies of scale, pre-treatment (biomass grinding and drying) accounts for 21.4% of the overall installed equipment cost, while product recovery accounts for 17.1% of the TIEC. The result for product recovery reported by Dutta et al. [47] accounted for 11.4% of the TIEC. This agrees with the results reported by van Schalkwyk et al. [46], where pre-treatment contributed between 15.4 and 21.4% of the TIEC and product recovery contributed 17.1% of the TIEC.

#### 3.4.2 Economic sensitivity analysis

**Fig. 7:**
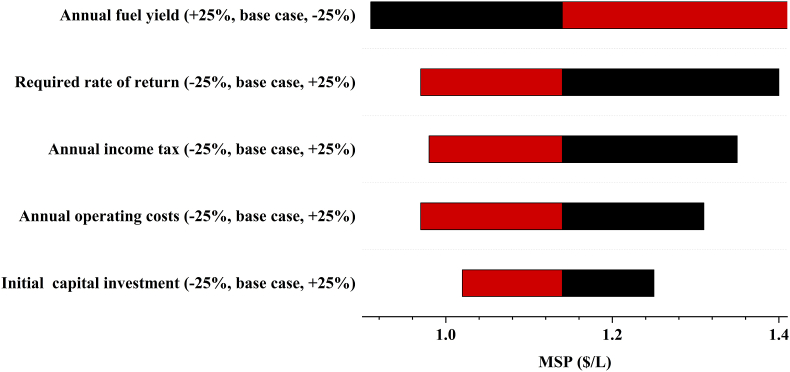
Economic sensitivity analysis bio-oil production.

The economic sensitivity analysis given in Fig. 7 assesses the impact of a 25% change in the economic parameters of bio-oil production. The MSP shows the highest sensitivity to variations in annual fuel yield, required rate of return, annual income tax, annual operating costs and initial capital investment. Carrasco et al. [48] and van Schalkwyk et al. [46] also found that the MSP of the liquid product is sensitive to a variation in the initial capital investment. The MSP of bio-oil was estimated at $1.14/L. This agrees with a value of $1.11/L obtained by Li et al. [49] in an analysis of biomass pyrolysis for the production of biofuels. A 25% increase in the required rate of return, annual income tax, annual operating costs and initial capital investment increases the MSP of bio-oil to $1.40/L, $1.35/L, $1.31/L and $1.25/L, respectively. However, increasing the annual fuel yield by 25% decreases the MSP to $0.91/L. Furthermore, a 25% reduction in the required rate of return, annual income tax, annual operating costs and initial capital investment decreases the MSP to $0.97/L, $0.98/L, $0.97/L and $1.02/L, respectively. Correspondingly, for a 25% reduction in annual fuel yield, the MSP of bio-oil increases to $1.52/L.

## 4 Conclusion

The main aim of the study was to model the MAP of pine sawdust and to optimize its process variables. Aspen Plus® V11 was used to model the MAP of pine sawdust, and a CCD with RSM was employed in optimizing the process variables. The RSM findings showed that the generation of bio-oil was more influenced by operating temperature. Additionally, the findings showed that the output of bio-oil was more significantly influenced by linear and quadratic terms of the reaction temperature. The maximum yield of bio-oil (65.8 wt%) was achieved at the optimized condition. The optimized values of experimental variables were 550 °C and 1 atm for operating temperature and reactor pressure respectively. The pyrolysis product yield may be predicted using quadratic models created based on the regression analysis. Reactor pressure does not significantly influence the distribution of the end products, according to the quadratic model developed for the pyrolysis product yield. With an overall high determination coefficient (R^2^ = 0.9883), the developed quadratic model fits the data well to predict the response. A MSP of $1.14/L of bio-oil was estimated from the economic evaluation of the model. A sensitivity analysis revealed that the annual fuel yield, required rate of return, annual income tax, annual operating costs and initial capital investment have a significant impact on the MSP. Previously reported studies on bio-oil production via fast pyrolysis utilizes conventional heating, however, this study explores microwave heating for applications in fast pyrolysis and evaluated the process economics. As a result, it can be inferred that using the optimized process parameters may improve the pyrolysis process' competitiveness on an industrial scale due to its better product yields and improved sustainability in the biorefinery, as well as assure waste reduction.

## Author contribution statement

Denzel Christopher Makepa: Conceived and designed the experiments; Performed the experiments; Analyzed and interpreted the data; Wrote the paper.

Chido Hermes Chihobo: Conceived and designed the experiments; Analyzed and interpreted the data.

Walter Rutendo Ruziwa: Contributed reagents, materials, analysis tools or data.

Downmore Musademba: Contributed reagents, materials, analysis tools or data; Wrote the paper.

## Funding statement

This research did not receive any specific grant from funding agencies in the public, commercial, or not-for-profit sectors.

## Data availability statement

No data was used for the research described in the article.

## Declaration of interest’s statement

The authors declare no conflict of interest.
